# A novel hybrid method based on Cuckoo optimization algorithm and artificial neural network to forecast world's carbon dioxide emission

**DOI:** 10.1016/j.mex.2021.101310

**Published:** 2021-03-15

**Authors:** Sayyed Abdolmajid Jalaee, Alireza Shakibaei, Hossein Akbarifard, Hamid Reza Horry, Amin GhasemiNejad, Fateme Nazari Robati, Naeeme Amani zarin, Reza Derakhshani

**Affiliations:** a Department of Economics, Faculty of Management and Economics, Shahid Bahonar University of Kerman, Kerman, Iran; b Department of Geology, Shahid Bahonar University of Kerman, Kerman, Iran; c Department of Earth Sciences, Utrecht University, Utrecht, the Netherlands

**Keywords:** Global climate changes, Metaheuristic method, Optimization algorithm

## Abstract

This paper deals with the global energy consumption to forecast future projections based on primary energy, global oil, coal and natural gas consumption using a hybrid Cuckoo optimization algorithm and information of British Petroleum Company plc and BP Amoco plc. The Artificial Neural Network (ANN) has some significant disadvantages, such as training slowly, easiness to fall into local optimal point, and sensitivity of the initial weights and bias. To overcome the shortcomings, an improved ANN structure, that is optimized by the Cuckoo Optimization Algorithm (COA), is proposed in this paper (COANN). The performance of the COANN is evaluated with Mean Squared Error (MSE), Root Mean Squared Error (RMSE), Mean Absolute Error (MAE), and Correlation Coefficient (CC) between the output of the model and the actual dataset. Finally, CO_2_ emission in the world by 2050 is forecasted using COANN. The findings showed that COANN is a helpful and reliable tool for monitoring global warming. This proposed method will assist experts, policy planners and researchers who study greenhouse gases.

• The method can be used as a potential tool for policymakers and governments to make policy on global warming monitoring and control.
• The proposed method can play a key role in the global climate changes policies and can have a significant impact on the efficiency or inefficiency of government's intervention policies.

## Graphical abstract

**Figure:**
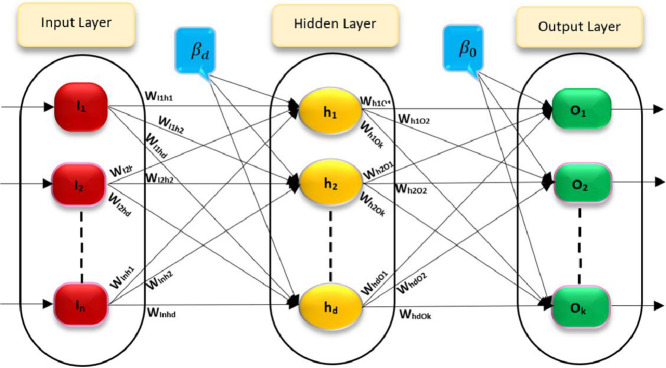

Specifications table

**Table:** 

Subject Area:	Environmental Science
More specific subject area:	Metaheuristic method, optimization methods, global warming, neural network
Method name:	COANN- a hybrid Cuckoo optimization algorithm with Artificial neural network
Name and reference of original method:	[1] Yang X-S, Deb S, editors. Cuckoo search via Lévy flights. 2009 World congress on nature & biologically inspired computing (NaBIC); 2009: IEEE. 10.1109/NABIC.2009.5393690 [2] Rajabioun R. Cuckoo optimization algorithm. Applied soft computing. 2011;11(8):5508–18. 10.1016/j.asoc.2011.05.008 [3] McCulloch WS, Pitts W. A logical calculus of the ideas immanent in nervous activity. The bulletin of mathematical biophysics. 1943;5(4):115–33. 10.1007/BF02478259
Resource availability:	[4] bp Energy Outlook, 2020 edition http://www.bp.com/energyoutlook

## Introduction

The use of non-classical methods to identify and predict complex systems problems has been expanded [5]. There are many methods to predict natural phenomena around the world, but it is still difficult to accurately forecast the events. In the real world, there are a huge amount of non-linear systems, or those whose behavior is dynamic and depends on their current state [6].

This paper introduces an objective approach for predicting world's carbon dioxide emission. The data of the global energy consumption are investigated for analyzing and forecasting the world's carbon dioxide emission using a hybrid method based on Cuckoo Optimization Algorithm and Artificial Neural Network (COANN).

## Methods and material

### Cuckoo Optimization Algorithm

The Cuckoo Optimization Algorithm is a new algorithm for solving nonlinear optimization problems with continuous variables. This algorithm was proposed by Yang and Deb [1], developed by Rajabioun [2] and inspired by the lifestyle of a bird called the Cuckoo. This algorithm runs like any other evolutionary algorithm. Cuckoos choose other birds’ nests to lay their eggs. They lay eggs similar to the eggs of the host bird in the nest and therefore use other birds to regenerate. Meanwhile, Cuckoo eggs may be recognized and destroyed by the host bird. Under such conditions, the Cuckoo migrates to areas that are more suitable for spawning and regeneration.

Like other Evolutionary Algorithms (EAs), this algorithm starts with an initial population, and like the Genetic Algorithm (GA), has a population-based approach. This population is made up of a number of Cuckoos that live in an area that is actually a decision-making space. A number of eggs are randomly assigned to each of these Cuckoos and each Cuckoo lays its eggs in a specific area. In each iteration, the value of the [*f* (habitat function)] is calculated for the habitat (equivalent to the individuals in GA) of each Cuckoo and its eggs. A number of these eggs (about 10%) that have a more unfavorable target function are identified and killed. If the best objective function of the remaining population satisfies the hold-up problem conditions, the solution process ends. Otherwise, the remaining eggs turn into adult Cuckoos and each of them migrates to the nearest and most suitable place [7].

This migration is represented using the following relation:

(1) $$Pop_{i}^{new}=Pop_{i}^{current}+\beta \left(Pop^{best}-Pop_{i}^{current}\right)$$

Where *Pop_i_^new^* is the ith new solution; *Pop_i_^current^* is the current ith solution; *b* is the movement coefficient, and *Pop^best^* is optimum current solution.

After migrating, the Cuckoos lay their eggs in their new habitat. Each Cuckoo is able to lay eggs in a specific radius, which is greater than the Egg-Laying Radius (ELR).

(2) $$ELR=\alpha \times \frac{N}{T}\times \left(Var_{hi}-\;Var_{low}\right)$$

Where α is an integer, which regulates the maximum value of ELR; N is the number of current eggs per Cuckoo; T is the total number of eggs; *Var_hi_* is the upper limit, and *Var_low_* is the lower limit of the decision variables. This process continues through an iterative process to find the best place to lay eggs, which is the optimal answer to the problem [8].

### Artificial Neural Network (ANN)

Artificial Neural Network (ANN) has been modeled using biological neural systems (the human brain). Although it processes information, it is relatively simpler than the human brain and easy to work with. ANNs are models for information processing that are generated by imitating biological neural networks such as the human brain. It is worth noting here that an important element of this model is the new structure of the information processing system, which includes a large number of elements (neurons) with strong internal connections, which work together to solve some specific problems. In this regard, the nature of neural network learning is of particular importance. Such networks, represented as learning systems, have the ability to learn from the past, experience, and environment, so that they can improve their behavior during any learning process. Network learning improvement is measured based on a criterion over time, in which this improvement criterion can model the purpose of the learning system.

In the above model, both processing and simulation are performed based on teaching and learning the relationships of the variables observed in the past, as well as the expansion of these relationships for the future to make some new predictions. It should be mentioned that the structure of this network consists of at least three layers of inputs, hidden, and output. The number of neurons in the input layer depends on the number of inputs. In this way, the input, hidden, and output layers, together with the neurons in each layer, form the network structure. The number of neurons in this layer, as well as the number of hidden layers depending on the type of problem is obtained using trial and error. The connection and relationship of the neurons of the inputs, hidden, and output layers are established by the weights, which are considered as the neural connections [9].

**Fig. 1:**
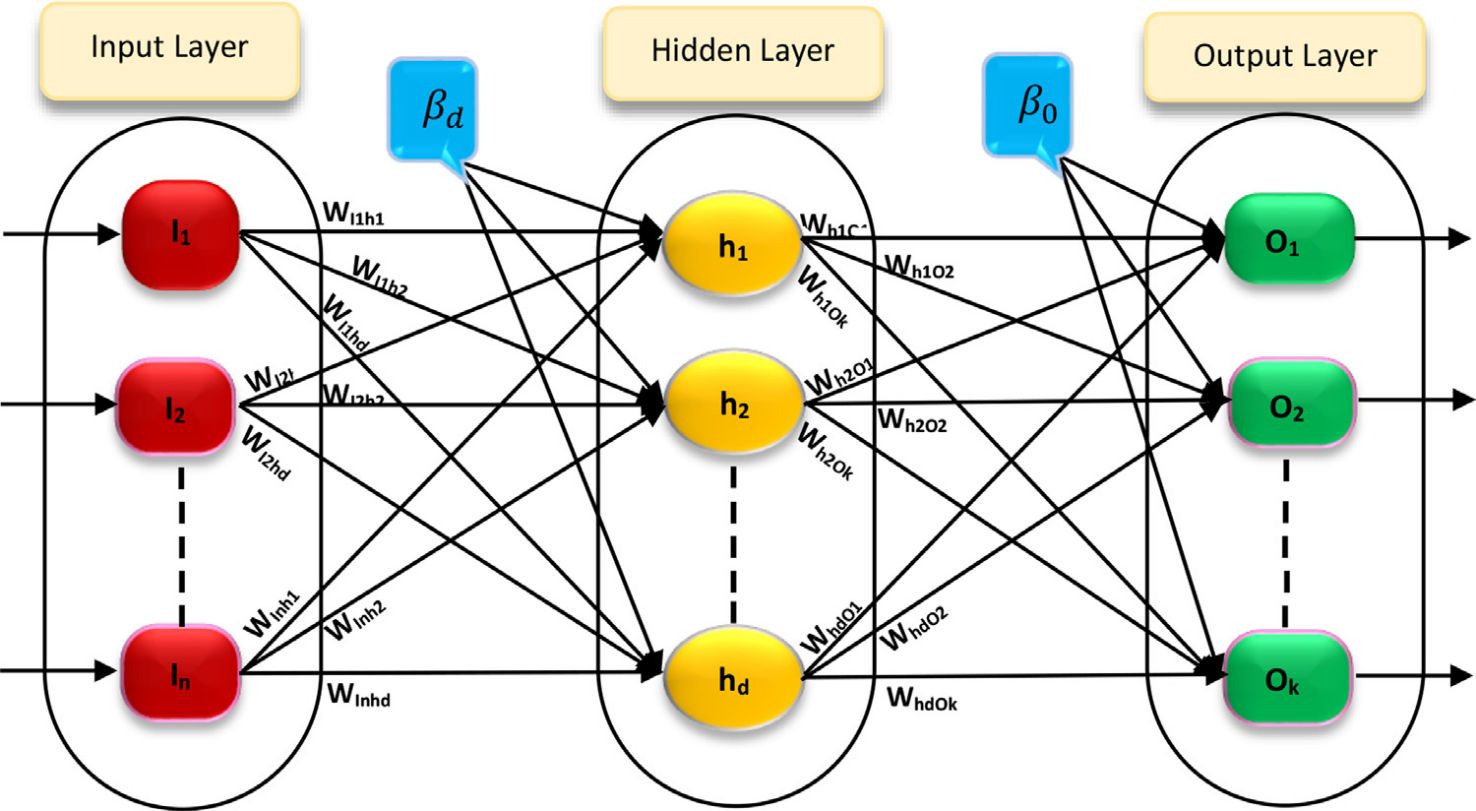
A three-layer artificial neural network.

The number of neurons in the input and output layers is determined by the problem for which the network is utilized. On the other hand, in terms of the hidden layer, the number of neurons is determined by the user using the trial-and-error method. In this regard, neural network training is one of the most important steps in the development of neural network model in which the network weight is determined under a nonlinear optimization process. Network learning is performed when the connection weights between the layers change so that the difference between the predicted and calculated values ​​is acceptable [3,10]. By achieving these conditions, the learning process is accomplished. Fig. 1 shows an ANN with a hidden layer, which contains some weights connecting between layers.

### Proposed method

Once a network is designed for a specific application, it is ready for training. In this paper, the Cuckoo Optimization Algorithm is used to search for a combination of weights and biases that provides the least error. The COA-optimized ANN method is divided into three parts: determining the structure of the ANN, obtaining the simplest weights and bias through COA, and predicting through the artificial neural network. It is decided that the ANN structure will support the value of input and output parameters in the first part, then the length of each Cuckoo in the COA is determined accordingly. In the second part, the COA method is used to optimize the weights and bias of the ANN. Each individual within the Cuckoo population includes all the weights and biases in ANN, and it's evaluated by the fitness function. The COA method implements initializing COA, determining fitness function, updating position operator, selecting operator, replacing operator, and eliminating operator to seek out the Cuckoo individual with the simplest fitness. This optimization process is repeated until satisfactory weights and bias are found. In the last part, the ANN with the optimal weights and bias is made and is trained to predict the output.

**Fig. 2:**
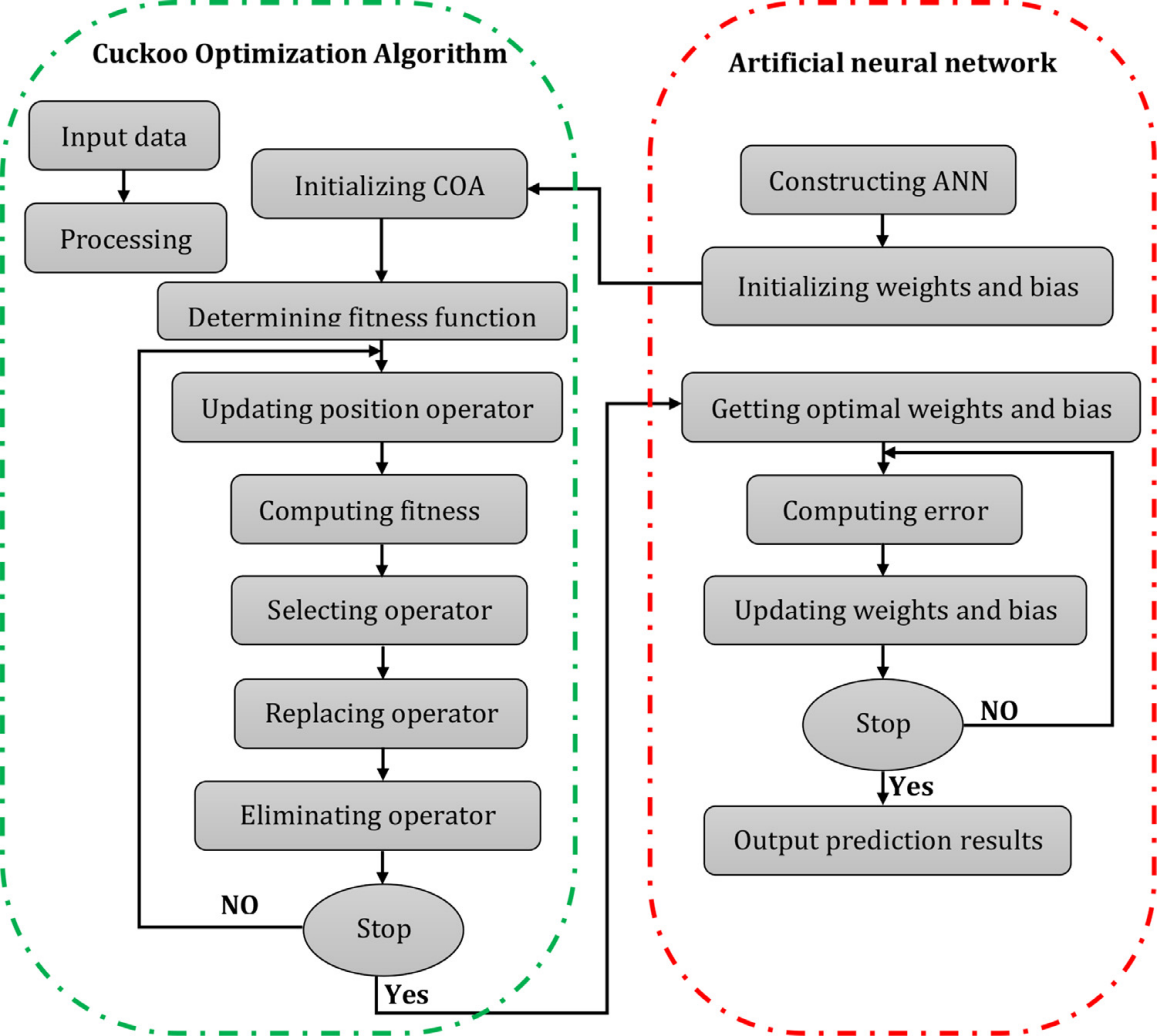
Flowchart of the COANN.

The Cuckoo optimization algorithm is utilized in COANN for optimizing the initial weights and artificial neural network's bias. Thus, the ANN predicts better output. The COANN elements are as follows: initializing COA, determining fitness function, updating position operator, selecting the operator, replacing the operator, and eliminating the operator to find the Cuckoo with the best fitness. The steps of the Cuckoo optimization algorithm are shown in detail in Figure 2.

(1) **Initializing COA:** The Cuckoo's individual encoding is done in real-coded form, and each individual consists of real number strings consisting of four sections: connection weights between output and hidden layers, connection weights between the input and hidden layers, the output layer's bias, and the hidden layer. All the weights bias artificial neural network are contained in each cuckoo. It is possible to construct a specific artificial neural network based on the bias and weights in ANN.
(2) **Determining Fitness Function:** Based on the best one, it is possible to specify bias and initial weights of ANN. When the artificial neural network is trained, it is utilized for output prediction.
(3) **Updating Position Operator:** The selection of a Cuckoo in the Cuckoo population is done randomly and according to (1), its position is updated. (3) is used for evaluating the ith cuckoo fitness at position xi(t) and generation t.
(4) **Selecting Operator:** In a similar way, selection of the other Cuckoo in the Cuckoo population is done randomly, and (3) is used for evaluating its position fitness of the ith Cuckoo at position (t) and generation t.
(5) **Replacing Operator:** If Fi>Fj, i.e. i.e., the Cuckoo i has a larger fitness value compared to the Cuckoo j, xj is substituted by the new solution.
(6) **Eliminating Operator:** To achieve the desired state for the population at all times, ceil(n*pa) the worst Cuckoos in each generation are eliminated. At the same time, to prevent changes in population size, ceil(n*pa) Cuckoos are randomly generated. Cuckoos with the best fitness are directly passed to the next generation. Ceil(x) here rounds the x elements to the closest integers towards infinity.

### Optimization and method validation

This paper presents a new method for forecasting global carbon dioxide emissions. Data on primary energy, global oil, coal, and natural gas consumption were collected a during the years 1980–2018 [4]. The input parameters of the neural network included primary energy, global oil, coal, natural gas consumption and the carbon dioxide emissions in the world were considered as the output parameters on the network. Data related to these parameters were divided into training, testing and data validation. 70% of these data was used for training, 15% for validation and another 15% for testing.

To estimate global carbon dioxide emission, it is necessary to normalize the data in the first step (Table 1). Eq. (3) was used to normalize the data.

(3) $$\;X_{N}=\left(X_{R}-X_{min}\right)/\left(X_{max}-X_{min}\right)$$

**Table 1:** Minimum and maximum values of research variables for normalization

VARIABLE	maximum	minimum
global oil consumption (Million tons)	2841.4	4350.3
natural gas (Billion cubic meters)	1224.2	2897.5
coal (Million tons oil equivalent)	1793.3	3867
primary energy consumption (Million tons oil equivalent)	6507.4	12819.4
Global CO_2_ emission (Million tons of carbon dioxide)	17976.7	32799.9

Where $X_{N}$ represents the normalized data and $X_{R}$ represents the original data value.

A multilayer perceptron neural network consists of an input layer, a hidden layer and an output layer. The neural network has 4 neurons in the hidden layers and one neuron in the output layer. The performance of the COANN method is evaluated with Mean Squared Error (MSE), Mean Absolute Error (MAE), Root Mean Squared Error (RMSE), and correlation coefficient (R) between COANN output and the actual dataset [7]. These errors are specified in the forms of Eq. (4)–(6)

(4) $$MSE=\frac{1}{n}\sum_{i=1}^{n}{\left(y_{\text{observed}}^{i}-y_{\text{predicted}}^{i}\right)}^{2}$$

(5) $$MAE=\frac{1}{n}\sum_{i=1}^{n}\left|y_{\text{observed}}^{i}-y_{\text{predicted}}^{i}\right|\;$$

(6) $$\text{RMSE}=\sqrt{\frac{1}{n}\sum_{i=1}^{n}{\left(y_{\text{observed}}^{i}-y_{\text{predicted}}^{i}\right)}^{2}\;}$$

**Fig. 3:**
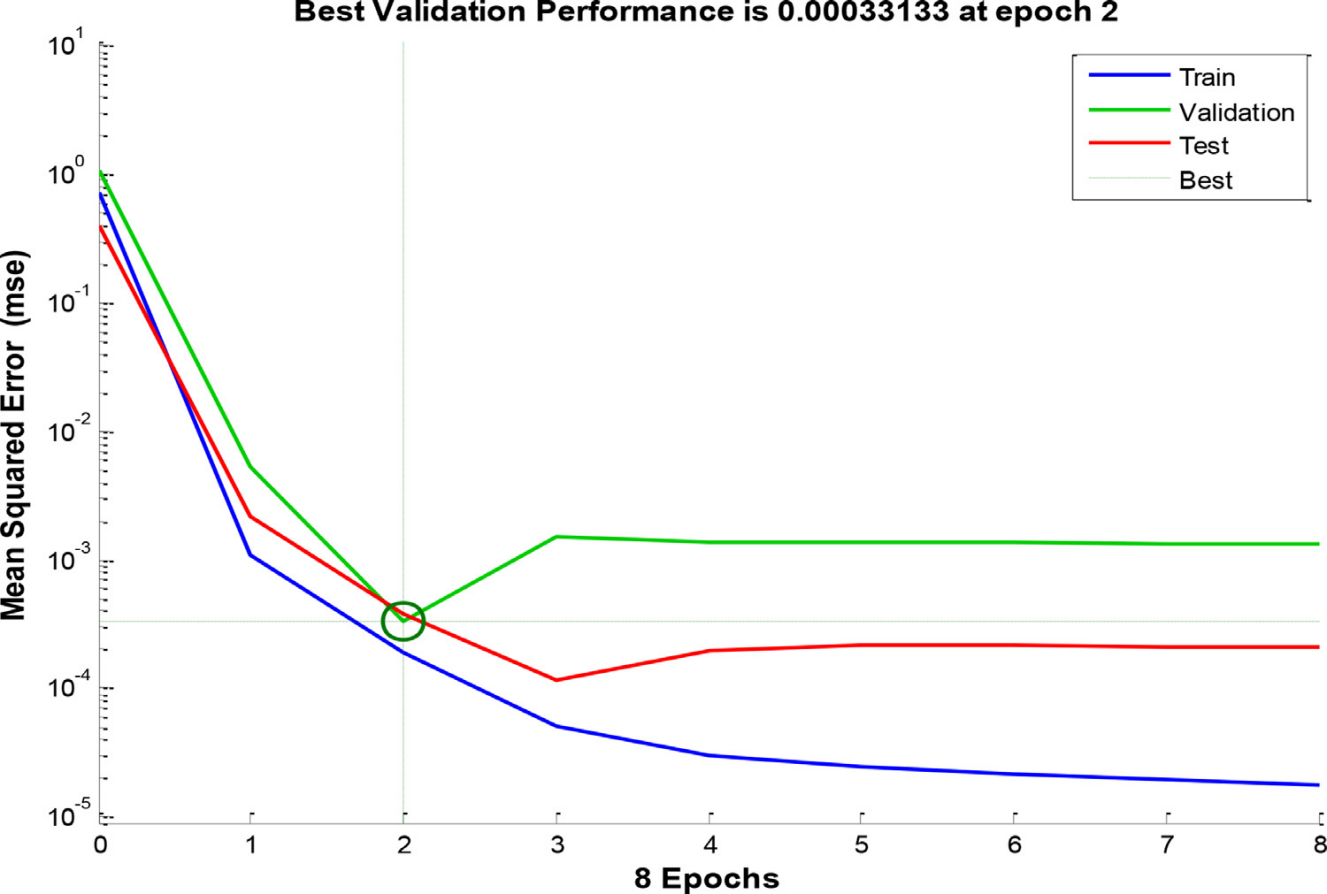
Performance Graph.

**Fig. 4:**
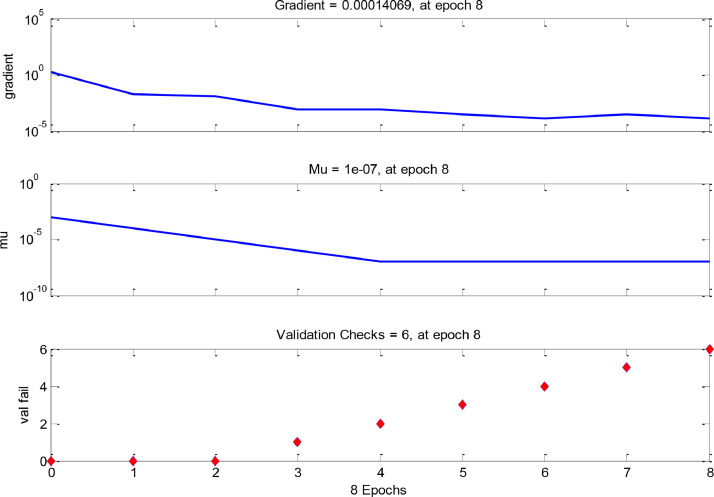
Optimized COANN output and model performance criteria for test data.

**Fig. 5:**
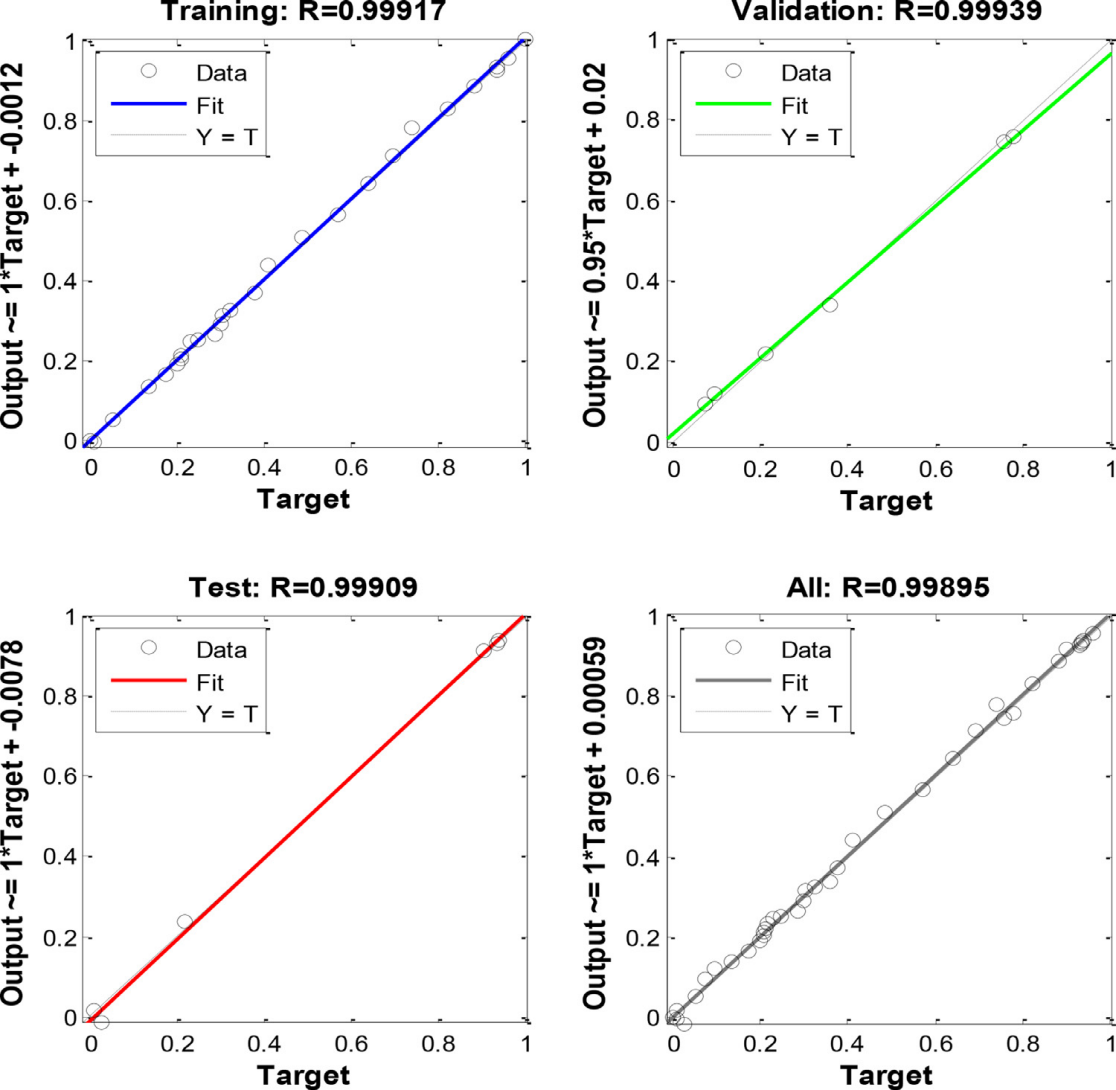
Regression Plot.

**Fig. 6:**
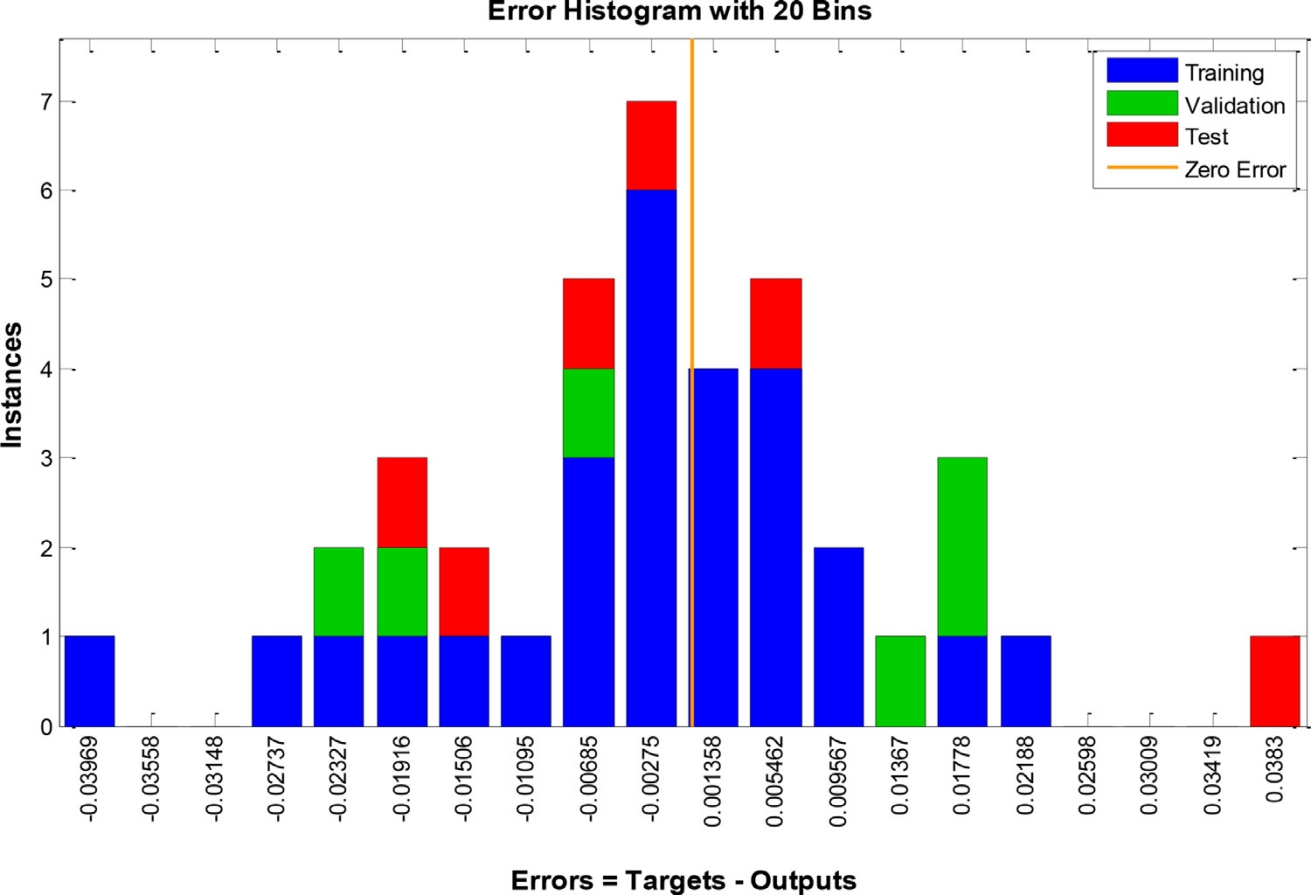
Error Histogram for COANN outputs.

Figs. 3, 4 and 5 indicate the best validation performance graph and regression plot between actual and predicted data in COANN method. Table 2 shows the performance evaluation of COANN outputs. Fig. 6 represents error histogram and performance criterion of COANN outputs.

Table 3 shows the performance of the COANN method for the modeling and the testing data. The findings showed that the proposed method can play a key role in the policy of controlling carbon dioxide emissions in the world and can have a significant impact on the efficiency or inefficiency of governments policies [1,2].

Using the COANN method, as outlined above, global carbon dioxide emissions are predicted by 2050. Table 4 indicates a comparison between forecasted published by British Petroleum company, BP, and the COANN method.

**Table 2:** COANN operation.

Neural network process	sample	MSE	RMSE	MAE	STD error	Mean error	R
training	27	0.000189	0.0137	-	-	-	0.9991
validation	6	0.000331	0.0182	-	-	-	0.9993
testing	6	0.000376	0.0194	-	-	-	0.9990
Total	39	0.000240	0.0150	0.011	0.015	0.0024	0.9989

**Table 3:** Comparison of Actual world's carbon dioxide emission values and their predicted values with COANN.

Date	Actual data	$\text{COANN}-output_{\text{predicted}}$	Relative error %
1986	19507.6	19581.9	0.38087
1990	21290	21289.8	-0.00093
1994	21652.9	21693.3	0.18658
1998	22792.7	22796.3	0.01579
2002	24502	24565.2	0.25793
2006	29018.8	29021.1	0.00792
2010	31057.9	31084.6	0.08596
2014	32844.8	32868.3	0.07154
2018	33890.8	33900.6	0.02891
Average	-	-	0.11495

**Table 4:** A comparison of projection for the years (2020–2040).

world's carbon dioxide emission					
year	2020	2025	2030	2035	2040
COANN method	34242.6	34855.3	35205.7	35630.1	35902.5
BP (2019)	34295.9	34877.1	35200.1	35658.7	35907.7

## Conclusion

In this paper, we proposed a hybrid COANN based on the cuckoo optimization algorithm and the artificial neural network. The proposed method has been successfully used to estimate world's carbon dioxide emission. The method can be used as a potential tool for experts, policy planners and researchers who study greenhouse gases.

## Declaration of Competing Interest

The authors declare that they have no known competing financial interests or personal relationships that could have appeared to influence the work reported in this paper.
